# Psilocybin therapy for females with anorexia nervosa: a phase 1, open-label feasibility study

**DOI:** 10.1038/s41591-023-02455-9

**Published:** 2023-07-24

**Authors:** Stephanie Knatz Peck, Samantha Shao, Tessa Gruen, Kevin Yang, Alexandra Babakanian, Julie Trim, Daphna M. Finn, Walter H. Kaye

**Affiliations:** 1 Department of Psychiatry, Eating Disorder Treatment & Research Center, University of California, San Diego, San Diego, CA USA; 2 University of Michigan Medical School, Ann Arbor, MI USA

**Keywords:** Outcomes research, Psychiatric disorders

## Abstract

Anorexia nervosa (AN) is a deadly illness with no proven treatments to reverse core symptoms and no medications approved by the US Food and Drug Administration. Novel treatments are urgently needed to improve clinical outcomes. In this open-label feasibility study, 10 adult female participants (mean body mass index 19.7 kg m^−^^2^; s.d. 3.7) who met *Diagnostic and Statistical Manual of Mental Disorders*, Fifth Edition (DSM-5) criteria for AN or pAN (partial remission) were recruited to a study conducted at an academic clinical research institute. Participants received a single 25-mg dose of synthetic psilocybin in conjunction with psychological support. The primary aim was to assess safety, tolerability and feasibility at post-treatment by incidences and occurrences of adverse events (AEs) and clinically significant changes in electrocardiogram (ECG), laboratory tests, vital signs and suicidality. No clinically significant changes were observed in ECG, vital signs or suicidality. Two participants developed asymptomatic hypoglycemia at post-treatment, which resolved within 24 h. No other clinically significant changes were observed in laboratory values. All AEs were mild and transient in nature. Participants’ qualitative perceptions suggest that the treatment was acceptable for most participants. Results suggest that psilocybin therapy is safe, tolerable and acceptable for female AN, which is a promising finding given physiological dangers and problems with treatment engagement. ClinicalTrials.gov identifier NCT04661514.

## Abstract (web summary)

Phase 1 trial results demonstrate that psilocybin in conjunction with psychological support is safe, tolerable and deemed acceptable and therapeutically meaningful by female individuals with anorexia nervosa.

## Main

Anorexia nervosa (AN) is a costly and deadly mental illness, which is notoriously difficult to treat^1^. It is associated with substantial morbidity and mortality, including an elevated suicide rate and an 18-fold increase in mortality^2,3^. Despite its seriousness, there are no proven treatments for adult AN that reverse core symptoms and no approved pharmacological interventions^1^. As a result, estimates suggest that less than half of patients achieve recovery; relapse rates approach 50%; and approximately 20% of those with AN will develop a chronic course^3^. There have been minimal advancements in novel treatment strategies and stagnant outcomes over the past several decades, resulting in a ‘crisis in care’^4,5^. Novel and innovative treatments methods are urgently needed to improve treatment engagement and outcomes. One such avenue may be psilocybin therapy.

Psilocybin is a psychedelic molecule whose mechanism of action is thought to be mediated by serotonin 2A (5-HT_2A_)^6^ and is the main psychoactive compound in the *Psilocybe* genus of mushrooms^7^. Considerable evidence suggests that individuals with AN have altered brain serotonin (5-HT) function, altered function of the 5-HT_2A_ receptor and altered endogenous brain 5-HT secretion^8,9^, supporting the speculation that the 5-HT_2A_ effects of psilocybin might effect change in AN symptoms. Unlike other serotonergic medications requiring repeated administrations, a single dose of psilocybin may lead to rapid and enduring synaptic adaptations that have the potential to improve AN symptoms^10^. However, the role of 5-HT dysfunction in AN and related affective states associated with restriction remains poorly understood with mixed findings^11,12^.

Mechanisms of action are not well elucidated, but psilocybin is thought to directly modulate the serotonergic system and indirectly modulate dopaminergic and glutamatergic systems and gene expression^10^. Psilocybin effects have been documented at the pharmacological, neural and psychological levels, all of which may contribute to improvements in mental illness^13^. Findings suggest that psilocybin may increase emotional and brain network plasticity, which may be responsible for sustained improvements in mental health status^14^. Psilocybin therapy typically involves the administration of psilocybin in conjunction with psychological support delivered by one to two trained therapists^15^. When administered in a safe and therapeutic setting in conjunction with psychological support, participants can report transformative experiences characterized by profound changes in values, beliefs and perspectives, which can lead to positive changes in subjective well-being, increased openness and greater cognitive flexibility^16–18^. Available evidence suggests that psilocybin therapy may hold promise for other treatment-resistant mental illnesses with studies demonstrating robust and rapid effects, but no modern studies have reported data on potential effects for AN^19^.

AN is characterized by excessive and undue preoccupation, fear and distress surrounding food, weight, shape and eating. This typically leads to rigid and repetitive behavioral patterns of control, such as restriction^20^. Improvements in anxiety^21^ and cognitive flexibility^18,22^, which have been shown to occur with psilocybin therapy, may assist with disrupting cardinal symptoms of AN mediated by these mechanisms, including eating disorder (ED)-related preoccupations, rigid thinking styles and entrenched behavioral patterns^23,24^. AN is often ego-syntonic in nature^25^, and AN behaviors are perceived as effective means to achieve internalized weight/shape ideals and avoid dysphoric mood states that result from eating^9^. Thus, individuals with AN may resist intervention or fail to acknowledge the seriousness of the illness, resulting in low treatment acceptability and substantial treatment dropout^26^. Psilocybin therapy, which has been shown to improve openness^27^ and occasion transformative experiences^17^, may facilitate a re-organization of values, shifting the relative importance of shape and weight and/or induce greater permeability to new attitudes and behaviors by directly targeting these features. Previous observational and naturalistic studies exploring the value of psilocybin and other psychedelic drugs in people with EDs have reported on emerging themes, such as increased affective and intellectual awareness, reduction in ED symptoms, positive mood changes, emotional processing and increases in self-acceptance^28–30^.

To date, no modern publications have reported data regarding the safety, tolerability and efficacy of psilocybin therapy for AN within the context of a clinical intervention; however, at the time of this publication, two additional registered clinical trials are currently underway (NCT04505189 and NCT04052568), and other ED expert groups have provided rationales for further evaluation of psychedelic treatments for AN^31,32^.

To our knowledge, the present study is the first modern trial to report data on the safety, tolerability and exploratory efficacy of a single 25-mg dose of psilocybin in conjunction with psychological support.

## Results

### Patient information

**Fig. 1:**
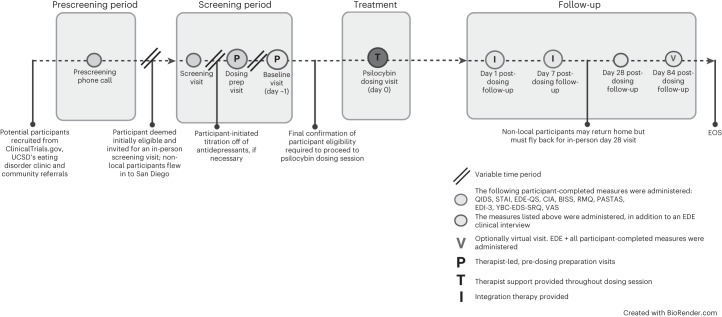
Study design and timeline. This figure outlines the participation process from prescreening through end of study (EOS) for participants.

**Fig. 2:**
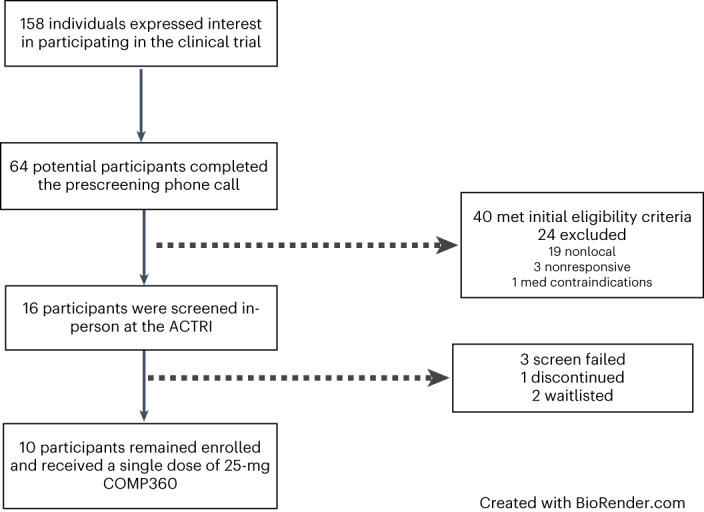
Participant screening flowchart. This figure summarizes the number of participants captured and retained through the screening process. COMP360, psilocybin. Source data

Enrollment started in April 2021 and finished in December 2021. In total, 158 individuals expressed interest in participating. Most were self-referred and became familiar with the study via ClinicalTrials.gov (ClinicalTrials.gov identifier: NCT04661514) or through community providers familiar with the study through community outreach efforts (see Table 1 for sample demographics, Fig. 1 for study design and timeline, and Fig. 2 for participant flowchart).

**Table 1:** Sample demographics

Demographic profile *n* = 10	Mean (s.d.)
Baseline BMI (kg m^−^^2^)	19.7 (3.7)
Duration of illness, years	8.9 (5.9)
Age, years	28.3 (3.7)
Ethnicity	
White/Caucasian	9 (90%)
Hispanic	1 (10%)
Diagnosis	
AN-R, current	4 (40%)
AN-BP, current	1 (10%)
AN-R, partial remission	5 (50%)
Gender	
Female	10 (100%)
Male	0 (0%)
Self-reported MDD comorbidity	7 (70%)
Self-reported GAD comorbidity	7 (70%)
Self-reported OCD comorbidity	3 (30%)
Prescribed serotonergic medications that require titration	7 (70%)

GAD, generalized anxiety disorder; OCD, obsessive compulsive disorder.

### Primary outcomes: safety and tolerability

To evaluate safety, we examined changes in vital signs, electrocardiograms (ECGs), clinical laboratory tests and suicidality from baseline (day −1) to day 1 (Table 2) and 1-week follow-up; we also examined reports of adverse events (AEs). The acute effects of psilocybin were well tolerated by all participants, and no serious AEs were observed (Table 2). No clinically significant changes were observed in vital signs or ECG. In relation to clinical laboratory values, two participants, both of whom were required to eat breakfast at the clinic before administration, developed hypoglycemia in follow-up clinical laboratory assessments, which resolved within 24 h. Participants were asymptomatic, and no intervention was required. No other clinically significant changes were observed in laboratory values. Suicidality was assessed using the Columbia Suicide Severity Rating Scale (C-SSRS)^33^ at each timepoint. There were no increases in suicidal ideation (SI), and no suicidal behaviors were present in the post-dosing follow-up visits. One participant with a history of major depressive disorder (MDD) and SI reported an increase in SI at 3-month follow-up, which did not appear related to study participation. AEs (Table 2) were mild and transient in nature, with headache, nausea and fatigue being the most common.

**Table 2:** Safety and tolerability: treatment-emergent AEs and summary of safety measures at post-treatment (day 1)

Treatment-emergent AEs	
MedDRA preferred term	COMP360 25 mg *n* = 10 (%)
Headache	8 (80%)
Fatigue	7 (70%)
Nausea	3 (30%)
Feeling abnormal	2 (20%)
Migraine	2 (20%)
Dizziness	2 (20%)
Illusion	2 (20%)
Pain	1 (10%)
Anxiety	1 (10%)
Orthostatic heart rate response increased	1 (10%)
Abdominal pain upper	1 (10%)
Safety assessments	
Clinically significant changes in clinical laboratory tests	
Hypoglycemia	2 (20%)
Clinically significant changes in ECG	0 (0%)
Clinically significant changes in vital signs	0 (0%)
Clinically significant increases in C-SSRS	0 (0%)

COMP360, psilocybin.

### Secondary outcomes: changes in psychopathology

**Extended Data Fig. 1:**
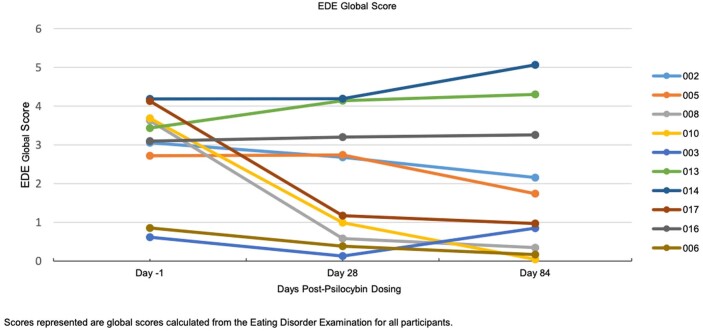
Eating Disorder Psychopathology over time, by Participant. Scores represented are global scores calculated from the Eating Disorder Examination for all participants. Source data

**Extended Data Fig. 2:**
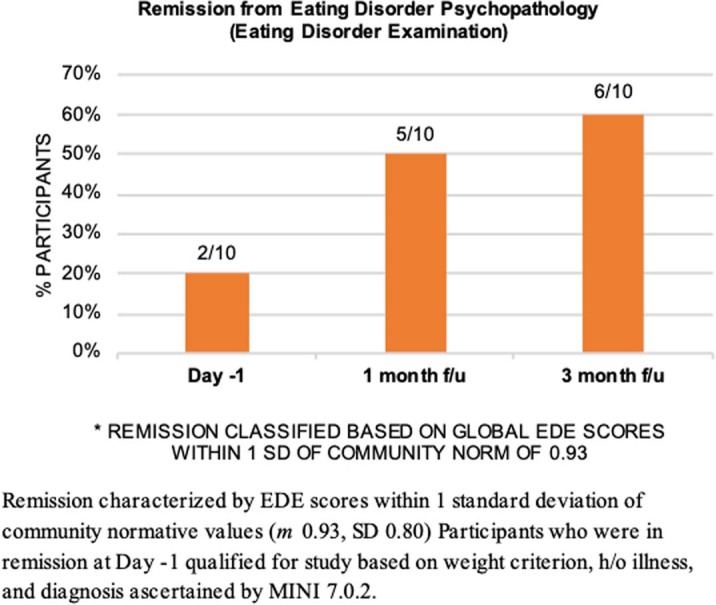
Number of participants with EDE scores within community norm values. * REMISSION CLASSIFIED BASED ON GLOBAL EDE. SCORES WITHIN 1 SD OF COMMUNITY NORM OF 0.93. Remission characterized by EDE scores within 1 standard deviation of community normative values (m 0.93, SD 0.805) Participants who were in remission at Day -1 qualified for study based on weight criterion, h/o illness, and diagnosis ascertained by MINI 7.0.2. Source data

Average changes on Eating Disorder Examination (EDE) subscales are shown in Table 3. Results from *t*-tests indicated that weight concerns decreased significantly from baseline (day −1) to 1-month (*P* = 0.036, Cohenʼs *d* = 0.78) and 3-month (*P* = 0.04, *d* = 0.78) follow-up, with a medium to large effect. Shape concerns significantly decreased at 1-month follow-up (*P* = 0.036, *d* = 0.78) but were no longer significant at 3-month follow-up (*P* = 0.081, *d* = 0.62). Changes on the eating concern and dietary restraint subscales were not significant, but changes in eating concerns approached significance at 3-month follow-up (*P* = 0.051, *d* = 0.71). Effects of treatment were, however, highly variable among participants, as is illustrated from the case series data (Extended Data Fig. 1). Four participants (40% of sample) demonstrated global EDE scores that decreased to within 1 s.d. of community norms (mean 0.93, s.d. 0.80)^34^ at 3-month follow-up (Extended Data Fig. 2), which we interpret to be clinically significant. No correlations were observed between any assessed participant characteristics and outcomes. Three of four responders met criteria for AN (versus pAN), and one had a diagnosis of anorexia nervosa binge–purge (AN-BP).

**Table 3:** EDE subscale scores over time and changes from baseline scores

*n* = 10	Visit	Mean (s.d.)	95% CI of mean difference	*P* value	Effect size (Cohen’s *d*)
Restraint	Day −1	2.18 (1.76)			
	1-month f/u	1.38 (1.56)	−1.81, 0.21	0.107	0.57
	3-month f/u	1.46 (1.84)	−1.81, 0.37	0.169	0.47
Eating concern	Day −1	2.10 (1.75)			
	1-month f/u	1.24 (1.50)	−1.83, 0.11	0.075	0.64
	3-month f/u	1.00 (1.32)	−2.20, 0.00	0.051	0.71
Shape concern	Day −1	3.68 (1.30)			
	1-month f/u	2.43 (1.84)	−2.40, −0.10	0.036	0.78
	3-month f/u	2.43 (1.95)	−2.69, 0.19	0.081	0.62
Weight concern	Day −1	3.64 (1.65)			
	1-month f/u	2.76 (1.81)	−1.69, −0.07	0.036	0.78
	3-month f/u	2.3 (2.00)	−2.57, −0.11	0.036	0.78

Subscales are calculated from the EDE. Day −1 is the day before psilocybin dosing session. Effect size was calculated using Cohen’s *d*. Analysis was performed using a two-sided 5% paired *t*-test with null hypothesis of no difference between baseline and post-baseline values.

CI, confidence interval; f/u, follow-up.

#### Body mass index

On average, changes in body mass index (BMI) were not statistically significant (see Extended Data Tables 2 and 3 for mean BMI scores over time and BMI changes over time for each participant). Of the four participants who demonstrated clinically significant reductions in eating disorder pathology as measured by the EDE, changes in BMI were variable, and there was no correlation between outcomes and weight status. Five participants demonstrated an increase in BMI at 3-month follow-up (range, 0.4–1.2 kg m^−^^2^).

#### Other secondary measures of psychopathology

Results for other secondary measures were also highly variable among participants. On average, participants demonstrated significant reductions at the primary endpoint of 1-month follow-up across the following domains: trait body image anxiety (Physical Appearance State and Trait Anxiety Scale (PASTAS)) (*P* = 0.04, *d* = 0.76), trait anxiety (Spielberger State-Trait Anxiety Inventory (STAI-T)) (*P* = 0.036, *d* = 0.78) and preoccupations and rituals surrounding food, eating and shape (Yale-Brown-Cornell Eating Disorder Scale (YBC-EDS)) (*P* = 0.043, *d* = 0.75) (Extended Data Tables 2 and 4).

### Exploratory outcomes: changes in psychopathology

#### Patient experience and acceptability

Qualitative responses highlighting participants’ reports of impactfulness are summarized in Table 4. Overall, the psilocybin experience was regarded as meaningful by participants. Ninety percent endorsed feeling more positive about life endeavors; 80% endorsed the experience as one of the top five most meaningful of life; and 70% reported experiencing a shift in personal identity and overall quality of life. Notably, 90% of participants reported that one dosing session was not enough. Phenomenological and qualitative information related to participants’ experience will be presented in a forthcoming manuscript.

**Table 4:** Qualitative perceptions of treatment

3-month follow-up % agreement since the psilocybin dosing…	*n* = 10
1. Have you felt that the overall quality of your life has improved?	70%
2. Have you felt as though the importance you place on your physical appearance has decreased?	60%
3. Have you felt more optimistic regarding your life endeavors?	90%
4. Have you felt a shift in your personal identity or a sense of who you are?	70%
5. Have you felt a greater sense of spirituality?	60%
6. Do you feel that the psilocybin dosing was one of the top five most meaningful experiences of your life?	80%
7. Was one dosing session enough? (% disagreement)	90% (No)

#### Response to psilocybin

**Extended Data Fig. 3:**
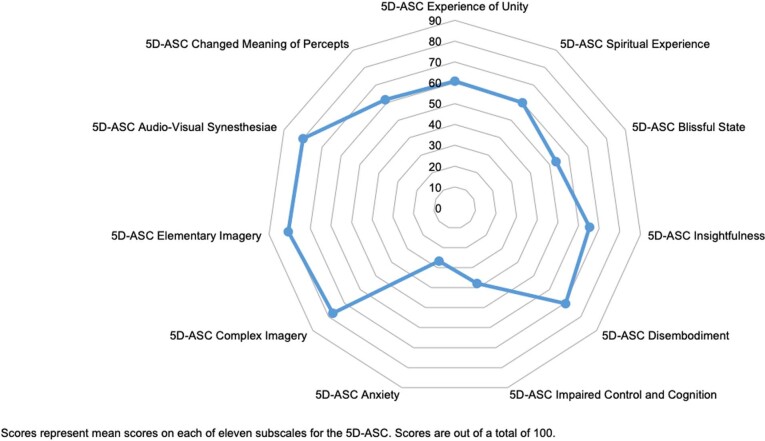
5-Dimensional Altered States of Consciousness (5D-ASC) Self-Report Ratings. Scores represent mean scores on each of eleven subscales for the 5D-ASC. Scores are out of a total of 100. Source data

Average self-reported intensity of the experience using the 11 subscales of the Five-Dimensional Altered States of Consciousness (5D-ASC) questionnaire is reported in Extended Data Fig. 3. No participants required anxiolytic rescue medication during the dosing session.

#### Exploratory measures of psychopathology

We also explored changes in depression scores (Quick Inventory of Depressive Symptomatology (QIDS)), functional impairment related to ED psychopathology (Clinical Impairment Assessment (CIA)) and readiness and motivation to change (Readiness Motivation Questionnaire (RMQ)) at 1-month follow-up. Results are presented in Extended Data Table 5. Results from *t*-tests indicated that functional impairment related to disordered eating as measured by the CIA decreased significantly from baseline (day −1) to 1 month (*P* = 0.041, *d* = 0.75). Other changes measured were not statistically significant.

## Discussion

To our knowledge, this is the first data report on the effects of psilocybin therapy in AN in a clinical research trial. This open-label pilot study examined the safety and tolerability of administering psilocybin therapy to participants with AN and pAN. We chose to include participants in partial remission because we were most interested in exploring potential changes in core ED psychopathology (versus weight), which can lead to treatment resistance and which can persist after weight restoration^35^. Additionally, pAN has been shown to be common, severe, persistent and undertreated^3,35^.

Psilocybin therapy, which includes psychological support by trained therapists, was found to be safe and well tolerated for the 10 participants who received treatment in this study. Most participants endorsed the treatment as highly meaningful and the experience as a positive life impact, and yet effects on ED psychopathology were highly variable, with a subset of participants demonstrating a robust response for a single-dose treatment. Results of this study are preliminary and inconclusive given its size and design. In this section, we discuss study findings related to primary outcomes, patient acceptability and qualitative perceptions as well as ED-specific psychopathology.

No participants in our study experienced any serious AEs, and all treatment-emergent AEs resolved within 24 h and without intervention (with the exception of one report of orthostatic heart rate that was reported at 3-month follow-up for one participant). Hypoglycemia occurred in two participants on the dosing day and resolved in 24 h. We hypothesize that this was related to a prolonged period of fasting on the dosing day (a common effect of psilocybin) versus any direct relationship to the drug, given the state of malnutrition and low carbohydrate stores associated with AN. Food was available on request to participants during the dosing day, but participants were not required to eat during the therapeutic experience. To our knowledge, there are no reports of psilocybin-induced hypogylcemia. However, individuals with AN often have reduced plasma glucose levels^36^. The incidence of hypoglycemia is clinically important and may indicate that attention to blood glucose levels before and after intervention may be warranted in participants with compromised nutritional status given the dangers associated with hypoglycemia in AN, including sudden death^36^. Both incidents of hypoglycemia occurred in participants who were given a standardized breakfast upon arrival. AN has a high prevalence of serious medical complications and physiological disturbances, which account for much illness-related death. The lack of negative incidences regarding safety in our study is promising for future research with the AN population; however, larger studies with a more diverse participant group continue to be needed to determine safety^37^.

Most participants self-reported positive changes 3 months after the psilocybin dosing. That the treatment was regarded as beneficial by most participants and that there were no dropouts are promising signs of engagement. Dropout rates in currently available treatments tend to be high^38^. Positive patient perceptions are also notable because they may suggest improved quality of life, which is clinically important for those with a potentially severe and enduring illness^38^. These self-reported data suggest that most participants perceived some benefit that may have been ED-non-specific in nature or not well captured by our assessment measures. Although our treatment included AN-specific therapeutic elements, adjunctive therapy was brief. Additional therapeutic methods, or extended therapeutic time, may be a useful consideration to facilitate further behavioral change and increased specificity, as has been employed in other psilocybin studies^39,40^. Our effect sizes and response rates were less robust than those reported for primary outcomes in psilocybin studies for other psychiatric disorders^41–43^. This may also be related to a heterogenous sample, a single-dose trial (compared to a two-dose design used in other studies), a lack of sensitivity in assessment measures or unique/specific features of AN that are not as readily addressed by psilocybin therapy or that require dosing adjustments. AN is a difficult-to-treat disorder with a complex physiology and physical recovery needs that differentiate it from other mental illnesses. Notably, 90% of participants reported that one dosing session was not enough, suggesting that an additional psilocybin experience(s) may be beneficial^44^.

Our exploratory analysis showed some significant reductions in ED-related psychopathology when the sample was aggregated; however, the results were highly variable among participants. Forty percent (4/10) of participants demonstrated clinically significant reductions^34^ in ED psychopathology (EDE) at 3-month follow-up, with scores decreasing from clinical ranges to within 1 s.d. of community normative values^34^. Within the responder group, ED psychopathology decreased precipitously and dropped below clinical levels within the month after the psilocybin dosing session. Three of the responders were not enrolled in any concurrent mental health services during the study period but had mental health treatment histories, and one was in longstanding, outpatient therapy that did not change during study enrollment. Symptoms continued to improve between 1-month and 3-month follow-up. Given the size and design of this study, these effects are preliminary and inconclusive. However, it is notable that we found such a robust response in a subset of participants in a single-dose trial of psilocybin delivered over a brief time period, because currently available treatments for adult AN result in only modest improvements in symptoms and often focus on weight and nutritional rehabilitation without adequately addressing underlying psychopathology^38^. Participants also experienced significant reductions in anxiety; however, mean changes in depression scores were not significant. Changes in general psychopathology may partially explain the effects on ED symptoms^44^.

We did not observe a significant effect on BMI over time, and results were highly variable among participants. BMI did not follow the same change trajectory as ED psychopathology for participants who showed reductions on core psychopathology. Of the four treatment responders, two showed positive BMI trends, one remained stable at a normal BMI and one showed a two-point decrease over time. It is also possible that a longer follow-up period is necessary to observe meaningful changes in BMI, which has been suggested by ED experts^45,46^. Notably, there are well-documented phenomena associated with AN that impede upon weight rehabilitation, including hypermetabolism and unusually high caloric requirements^37^. When queried about the lack of weight change, one treatment responder stated, ‘The irony is that now that I want to recover I can eat intuitively, but that is not enough to support physical recovery’. These findings may suggest that targeted nutritional rehabilitation emblematic of traditional treatment may be a necessary adjunctive treatment even when significant improvements in ED psychopathology are conferred.

Limitations of this study include its small sample size, the lack of a control comparison and the open-label design. Owing to the exploratory nature of this study, we did not conduct a power analysis or correct for multiple comparisons. As a result, these findings are not conclusive and should be interpreted with caution. Additionally, all of the participants were self-referred, which may have resulted in a selection bias. Similarly, suggestibility and expectations of positive outcomes related to positive popular media coverage surrounding psychedelics, and attending treatment at a well-reputed ED service (particularly for those who were nonlocal), may have resulted in a suggestibility that influenced positive outcomes. Our sample included a diverse range of severity; however, many participants had mild to moderate AN. More research is needed to evaluate psilocybin therapy for severe presentations. The study also lacked gender, racial and cultural diversity.

Strengths of this study included administration of a precise dose of pure synthesized psilocybin and the evaluation of a highly novel treatment modality for a treatment-resistant population. Larger, adequately powered, well-controlled trials with comparator treatments are needed to evaluate the efficacy of psilocybin therapy in a diverse sample of patients with AN. Future studies should further investigate mechanisms of action and moderators of treatment to discern how psilocybin may lead to changes in AN and whether psilocybin therapy is more suitable and effective for a specific subset of AN. Additionally, future studies are needed for optimization to identify adequate dosage, identify the optimal number of psilocybin administrations and investigate the need for possible adjunctive treatments that could optimize treatment outcomes.

In conclusion, results from this open-label, single-arm study suggest that psilocybin therapy is safe and tolerable in participants with AN; however, adequately powered, randomized controlled trials are needed to draw any conclusions.

## Methods

### Study design and participants

This open-label pilot study evaluated the safety, tolerability and preliminary efficacy of psilocybin therapy for participants with AN. Participants were 10 female adults who met current *Diagnostic and Statistical Manual of Mental Disorders*, Fifth Edition (DSM-5) criteria for AN (anorexia nervosa restricting (AN-R) and AN-BP subtypes) within a current episode or in partial remission. All participants received a 25-mg dose of investigational drug COMP360, a proprietary pharmaceutical-grade synthetic psilocybin formulation, optimized for stability and purity, developed by COMPASS Pathfinder Ltd. administered in conjunction with psychological support. The inclusion criteria were a current diagnosis of AN or pAN and an age range of 18–40 years. No financial compensation was provided for participation. Medical exclusion criteria included BMI < 16 kg m^−^^2^, medical instability, positive pregnancy test, cardiovascular conditions within the last year and any other clinically significant illnesses or disturbances of physical systems. Psychiatric exclusions included a current or previously diagnosed psychotic disorder, substantial suicide risk, substance use disorder (within the last year), history of mania and positive screening for borderline personality disorder (McLean Screening Instrument). Demographics are reported in Table 1.

Study information was posted on ClinicalTrials.gov and advertised through the Eating Disorder Treatment & Research Center at the University of California, San Diego (UCSD). The trial was approved by the US Food and Drug Administration, the Regulatory Approval Committee of California and the UCSD Institutional Review Board (site-specific approvals). All participants provided written informed consent to the study team personnel at the start of the in-person screening visit. It is well known that AN is more prevalent and more often diagnosed in females^47,48^. Thus, it is challenging to recruit males. The vast majority of those who expressed interest in this study were female (<1% male), and all participants were identified females by self-report.

### Procedures

The study investigational drug was COMP360, a proprietary pharmaceutical-grade synthetic psilocybin formulation, optimized for stability and purity, developed by COMPASS Pathfinder Ltd. (five capsules of 5 mg of psilocybin)^49^. A DEA Form 223 license was obtained by the principal investigator (PI) for storage and dispensing authorization. Screening included review of informed consent and written signature and medical screening, including an ECG, blood tests, assessment of vitals and review of past medical records. This also included a psychiatric interview, and assessment, including the Mini-International Neuropsychiatric Interview 7.0.2 (MINI)^50^, to ascertain a diagnosis of AN and rule out a current or past psychotic disorder, bipolar disorder and history of mania was completed. Other psychiatric screening assessments included imminent suicidality within the past year (C-SSRS)^33^, the McClean Screening Instrument for Borderline Personality Disorder^51^ and an interview informed by DSM-5 criteria to rule out substance use disorders within the past year. Ten eligible participants were included in the study, including three participants from outside of the southern California region who received partial study funding for relocation expenses. Participants did not enroll/change mental health treatment during study screening and enrollment. One participant received concurrent treatment at a partial hospitalization program commencing at the onset of screening. Five participants continued to participate in ongoing weekly outpatient therapy services that did not change during study enrollment.

The study team reviewed the results of the screening to determine eligibility. Eligible participants who were on serotonergic medications were titrated off these medications over the screening period. During this ‘washout period’, they met with the study PI, who monitored titration and assessed response, and a clinical psychologist, who conducted a suicide assessment (C-SSRS) weekly throughout the screening period. Baseline assessments were conducted the day before the psilocybin session (day −1) (see Fig. 1 for study schemata, visit timeline and repeated assessments).

In the 2 weeks leading up to the psilocybin session, participants met with the lead study psychologist for two preparation sessions (Extended Data Table 1, ‘Overview of the therapeutic model’). All sessions were conducted at the UCSD Altman Clinical and Translational Research Institute (ACTRI). On the day of the dosing session, participants arrived at the clinic at 7:00. Upon arrival to the clinic, the study team assessed participant orthostatic vitals, and the participant completed a urine drug screen. We introduced a standardized breakfast at the sixth participant as a measure to control for hypoglycemia. After determining a negative screen and consuming food (for participants 6–10), the study team then administered 25 mg of psilocybin (five capsules × 5 mg). Two psychologists were present in the room throughout the day to provide support and assess for safety, and participants were required to remain in their rooms at the ACTRI for 8 h commencing from the time the psilocybin was ingested. Before departure and once a participant reported diminished effect of the drug and a return to baseline experience, the study team administered the 5D-ASC rating scale^52^, and the study PI determined the participant’s suitability to be discharged (Fig. 2).

Participants returned to the clinic the morning after the dosing visit (day 1), during which they completed post-treatment repeated assessments and met the study psychologists for a safety assessment and a 60–90-min integration session (see the ‘Inventory of Supporting Information’ document, ‘Overview of the therapeutic model’, for information on integration sessions). Participants then returned to the clinic for continued assessment at 1-week, 1-month and 3-month follow-up. Participants also received two integration sessions at day 1 and day 7. The 3-month follow-up visit was conducted via telehealth for seven participants owing to change in location.

### Assessments

The main objective of this study was to evaluate the safety and tolerability of psilocybin therapy for AN. To this end, the primary outcome measures were reports of AEs, changes in vital signs, ECGs, clinically significant changes in clinical laboratory tests and suicidality. These features were assessed in all 10 participants the day before the dosing session (day −1), after the dosing session (day 1) and at 1-week follow-up to evaluate any changes. We also assessed pre-specified secondary measures of response to the treatment and preliminary treatment efficacy by exploring changes in ED symptoms and behaviors and related psychopathology (*n* = 10 participants). The primary measure for exploring effects of the treatment on ED symptoms and behaviors was the EDE^53^. We explored sample mean changes in ED symptoms and behaviors, including weight concerns, shape concerns, eating concerns and dietary restraint at 1 month and 3 months after the dosing session.

We were primarily interested in changes in the EDE because it is designed to assess the full range of specific psychopathology related to EDs and is considered the gold standard measurement^53^ for ED pathology with good reliability and validity. We also administered the EDE-QS^54^, which is a self-report questionnaire form of the EDE (delivered at 1-week follow-up), and the Eating Disorders Inventory (EDI)^55^. Additionally, we assessed changes in features that can characterize AN, including BMI, body image (PASTAS)^56^, Body Image State Scales (BISS)^56,57^, state and trait anxiety (STAI-T)^58^ and preoccupation and rituals related to eating, food, weight and shape (YBC-EDS)^59^ (*n* = 10 participants).

Pre-specified exploratory outcomes included assessing for changes in ED-related functional impairment (CIA)^60^, depressive symptoms (Quick Inventory of Depressive Symptomatology-Self Report (QIDS-SR))^61,62^, readiness toward actionable change (Readiness and Motivation Questionnaire)^62^, psychedelic intensity ratings (5D-ASC)^52^ and patient acceptability. The primary endpoint for all psychological measures was 1-month follow-up. We also repeated measures at 3-month follow-up. At 3-month follow-up, patient acceptability was evaluated by asking participants qualitative yes/no questions about the effect of their experience on life and well-being, some of which were developed by Griffiths et al.^63^.

### Statistical analysis

Owing to the exploratory nature of this study, we did not perform a power analysis. Ten participants were studied to provide an initial impression of safety, tolerability, acceptability and efficacy of this treatment in an effort to inform future trials. In this report, we summarize the results of 10 participants by presenting incidences of AEs and incidences of significant changes in our safety measures (ECG, clinical laboratory tests, suicidality and vitals) at day 1 and 1-week follow-up. To explore initial efficacy, we evaluated mean sample changes in secondary outcome measures at 1-month follow-up using paired *t*-tests (α = 0.05, two-sided). All data were collected using Qualtrics (version dates April 2021, May 2021 and November 2021). Data were analyzed using SAS analytic software version 9.4 We also present individual participant changes in the EDE and BMI (primary diagnostic qualifiers)^64^ at 1-month and 3-month follow-up using a case series design. Owing to the small sample size for this initial pilot study, we have also included effect sizes using Cohen’s *d* values. Multiple a priori comparisons were explored for this study; however, owing to its exploratory nature, we chose not to use a correction accounting for multiple comparisons.

### Reporting summary

Further information on research design is available in the Nature Portfolio Reporting Summary linked to this article.

## Online content

Any methods, additional references, Nature Portfolio reporting summaries, source data, extended data, supplementary information, acknowledgements, peer review information; details of author contributions and competing interests; and statements of data and code availability are available at 10.1038/s41591-023-02455-9.

### Supplementary information

Reporting Summary

### Source data

Source Data Fig. 2 Unidentified prescreen and screen information.

Source Data Extended Data Fig. 1 Global EDE scores at timepoints: day −1, day 28 and day 84.

Source Data Extended Data Fig. 2 Global EDE scores at timepoints: day −1, day 28 and day 84 (community normative values (mean/s.d.) noted on spreadsheet).

Source Data Extended Data Fig. 3 Participant subscale scores for 5D-ASC questionnaire.

Source Data Extended Data Table 2 Baseline (day −1) and day 28 (1 month) participant scores for EDE-QS, EDI, YBC-EDS and BMI assessments.

Source Data Extended Data Table 4 Baseline (day −1) and day 28 (1 month) participant scores for PASTAS-trait, PASTAS-state, STAI-state, STAI-trait and BISS.

Source Data Extended Data Table 5 Baseline (day −1) and day 28 (1 month) participant scores for CIA, QIDS and RMQ Action.

## Figures and Tables

**Extended Data Table 1:**
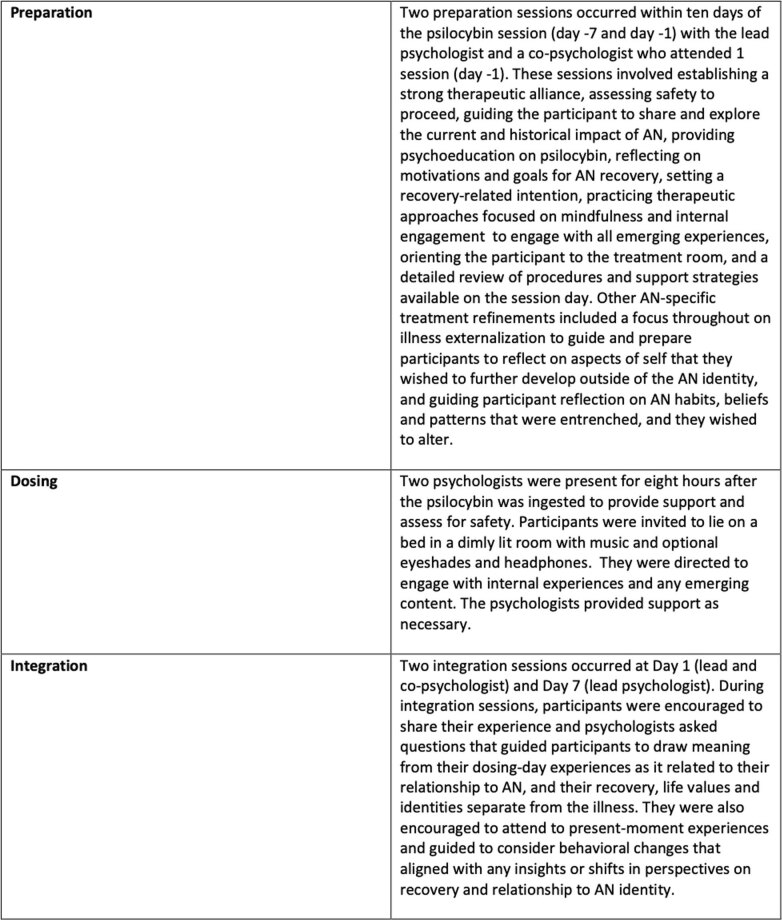
Overview of the therapeutic model

**Extended Data Table 2:**
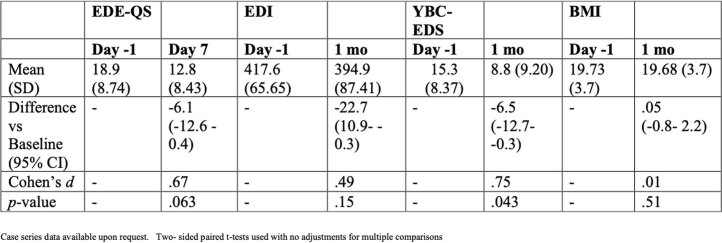
Secondary outcomes: clinical rating scores of ED psychopathology at baseline and 1-month follow-up

**Extended Data Table 3:**
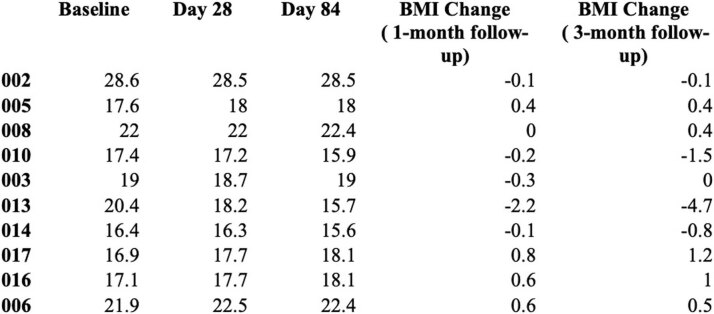
Participant BMI and BMI change over time

**Extended Data Table 4:**
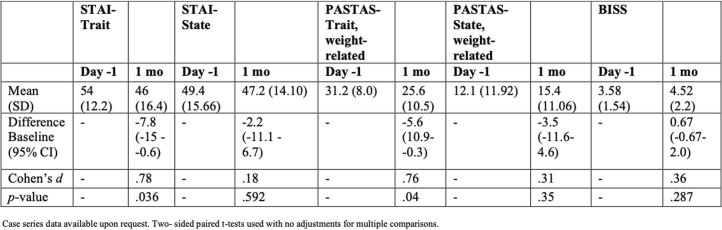
Secondary outcomes: clinical rating scores of psychopathology at baseline and 1-month follow-up

**Extended Data Table 5:**
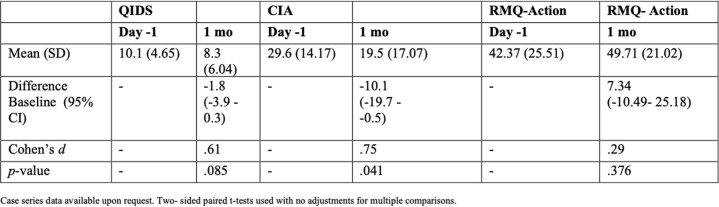
Exploratory outcomes: clinical rating scores of psychopathology at baseline and 1-month follow-up

## Data Availability

The datasets generated and/or analyzed during the current study are available in the Dryad Data repository and available upon reasonable request. Data include individual de-identified participant data and de-identified individual responses to self-report assessments, semi-structured interviews and physiological and biological data. Links to dataset are as follows: https://datadryad.org/stash/share/-l-cJuzYIJo3_ftcjtpYZgcZzIttyqQJf0AOTZRWbkM and 10.5061/dryad.47d7wm3hq. A summary of the research protocol is available on ClinicalTrials.gov (identifier: NCT04661514) and is available upon reasonable request. Source data are provided with this paper.
