# Supplementary material for: Psilocybin therapy for females with anorexia nervosa: a phase 1, open-label feasibility study
Source: Nat Med. 2023 Jul 24;29(8):1947–53. doi: 10.1038/s41591-023-02455-9 (PMC10427429; doi:10.1038/s41591-023-02455-9)
Supplement: Supplementary file 1 — Reporting Summary [file 41591_2023_2455_MOESM1_ESM.pdf]

nature portfolio

Corresponding author(s): Stephanie Knatz Peck, PHD
Walter Kaye, MD

Last updated by author(s): 06/01/2023

Reporting Summary

Nature Portfolio wishes to improve the reproducibility of the work that we publish. This form provides structure for consistency and transparency in reporting. For further information on Nature Portfolio policies, see our Editorial Policies and the Editorial Policy Checklist.

Statistics

For all statistical analyses, confirm that the following items are present in the figure legend, table legend, main text, or Methods section.

n/a	Confirmed	
☐	☒	The exact sample size (*n*) for each experimental group/condition, given as a discrete number and unit of measurement
☒	☐	A statement on whether measurements were taken from distinct samples or whether the same sample was measured repeatedly
☐	☒	The statistical test(s) used AND whether they are one- or two-sided<br>*Only common tests should be described solely by name; describe more complex techniques in the Methods section.*
☒	☐	A description of all covariates tested
☐	☒	A description of any assumptions or corrections, such as tests of normality and adjustment for multiple comparisons
☐	☒	A full description of the statistical parameters including central tendency (e.g. means) or other basic estimates (e.g. regression coefficient) AND variation (e.g. standard deviation) or associated estimates of uncertainty (e.g. confidence intervals)
☐	☒	For null hypothesis testing, the test statistic (e.g. *F*, *t*, *r*) with confidence intervals, effect sizes, degrees of freedom and *P* value noted<br>*Give P values as exact values whenever suitable.*
☒	☐	For Bayesian analysis, information on the choice of priors and Markov chain Monte Carlo settings
☒	☐	For hierarchical and complex designs, identification of the appropriate level for tests and full reporting of outcomes
☐	☒	Estimates of effect sizes (e.g. Cohen's *d*, Pearson's *r*), indicating how they were calculated

Our web collection on statistics for biologists contains articles on many of the points above.

Software and code

Policy information about availability of computer code

Data collection	Qualtrics software was used for data collection (version dates April 2021, May 2021, and November 2021).
Data analysis	SAS analytic software used for data analysis. Version: 9.4

For manuscripts utilizing custom algorithms or software that are central to the research but not yet described in published literature, software must be made available to editors and reviewers. We strongly encourage code deposition in a community repository (e.g. GitHub). See the Nature Portfolio guidelines for submitting code & software for further information.

Data

Policy information about availability of data

All manuscripts must include a data availability statement. This statement should provide the following information, where applicable:

- Accession codes, unique identifiers, or web links for publicly available datasets
- A description of any restrictions on data availability
- For clinical datasets or third party data, please ensure that the statement adheres to our policy

Data is available on Dryad https://datadryad.org/stash/share/-l-cJuzYlJo3_ftcjtpYGgcZzIttyqQJf0AOTZRWbkM DOI: doi:10.5061/dryad.47d7wm3hq

Human research participants

Policy information about studies involving human research participants and Sex and Gender in Research.

Reporting on sex and gender	Participants did not report on sex. All participants self-reported gender, which is described in the study. All participants identified as female.
Population characteristics	See above
Recruitment	Participants initiated referral for study in response to study information posted on ClinicalTrials.gov and study information posted on the UCSD Eating Disorder Treatment and Research Center website. Since participants self-initiated, it is possible that there is a selection bias that distinguishes the sample from the population, which may include those more motivated to engage in treatment, those with an interest in psychedelics (the majority of participants were psychedelic-naive), and those with potential expectancy effects abopout psychedelics.
Ethics oversight	The trial was approved by the Federal Drug Administration (FDA), the Regulatory Approval Committee of California (RAP-C), and the UC San Diego's Institutional Review Board (site specific approvals). All participants provided written informed consent to the study team personnel at the start of the in-person screening visit.

Note that full information on the approval of the study protocol must also be provided in the manuscript.

Field-specific reporting

Please select the one below that is the best fit for your research. If you are not sure, read the appropriate sections before making your selection.

☐ Life sciences ☒ Behavioural & social sciences ☐ Ecological, evolutionary & environmental sciences

For a reference copy of the document with all sections, see nature.com/documents/nr-reporting-summary-flat.pdf

Behavioural & social sciences study design

All studies must disclose on these points even when the disclosure is negative.

Study description	This is an open label feasibility study evaluating the safety, tolerability, and preliminary efficacy of a single dose of 25mg synthetic psilocybin with psychological support to a sample of 10 participants with Anorexia Nervosa . The results reported are quantitative. We report on changes in eating disorder psychopathology at 1 and 3 month follow up, in addition to adverse events, and safety measures.
Research sample	Participants were 10 females (self-reported gender) with a diagnosis of anorexia nervosa (AN), or anorexia nervosa, partial remission (pAN). The average age was 28.3 years, the average duration of illness was 8.9 years, and average baseline BMI was 19.7. These characteristics are representative of the population, however baseline BMI was slightly higher than the traditional cutoff of 18.5 (no longer used as diagnostic criteria). We chose to include pAN because participants had continued to endorse core AN psychopathology, and in some cases were nutritionally rehabilitated by continued to meet criteria for AN. Furthermore, pAN is associated with treatment resistance and severity.<br>The sample is representative of AN and pAN. It is well known that AN is more prevalent and more often diagnosed amongst females.
Sampling strategy	This was a convenience sample for this open label pilot study. We chose to study 10 participants to assess preliminary effects, safety, and tolerability because this is the first trial to report on psilocybin-assisted therapy for an AN sample. Results of this trial informed the design and progression of a future randomized controlled trial.
Data collection	The experimenters were not blinded to condition (open label). Participants completed assessments on an IPAD or laptop computer under the supervision of the research coordinator. No one else was present during completion of assessments.
Timing	Data collection started on 04/07/2021 and stopped on 06/07/2022.
Data exclusions	No data were excluded.
Non-participation	There were no drop-outs. One participant who was deemed eligible for enrollment and was screened declined participation due to concerns about washing off her serotanergic medications.
Randomization	Participants were not allocated into groups.

Reporting for specific materials, systems and methods

We require information from authors about some types of materials, experimental systems and methods used in many studies. Here, indicate whether each material, system or method listed is relevant to your study. If you are not sure if a list item applies to your research, read the appropriate section before selecting a response.

Materials & experimental systems

n/a	Involved in the study
☒	☐ Antibodies
☒	☐ Eukaryotic cell lines
☒	☐ Palaeontology and archaeology
☒	☐ Animals and other organisms
☐	☒ Clinical data
☒	☐ Dual use research of concern

Methods

n/a	Involved in the study
☒	☐ ChIP-seq
☒	☐ Flow cytometry
☒	☐ MRI-based neuroimaging

Clinical data

Policy information about clinical studies

All manuscripts should comply with the ICMJE guidelines for publication of clinical research and a completed CONSORT checklist must be included with all submissions.

Clinical trial registration	ClinicalTrials.gov Identifier: NCT04661514
Study protocol	Full study protocol submitted with initial submission
Data collection	Recruitment commenced in April of 2021 and was completed in December of 2021. Data collection began in April 2021 and continued through March 2022. The study and assessments were conduced at the Altman Clinical Translational Research Institute, located on the UCSD School of Medicine campus.
Outcomes	Primary and secondary outcomes measures were defined in the initial study protocol, and included safety data (changes in clinical labs, EKG, vitals and suicidality); tolerability (adverse events). The secondary outcomes were efficacy assessments measured using the Eating Disorder Examination, the gold standard assessment for eating disorder psychopathology.
